# The role risk of cigarette smoking, alcohol consumption, sleeping disorder, and mental health with hearing loss among stroke patients

**DOI:** 10.18332/tpc/210928

**Published:** 2025-11-03

**Authors:** Abdulbari Bener, Ahmet Erdoğan, Lima Oria, Hajira Karim, Lütfü Hanoğlu

**Affiliations:** 1 Department of Biostatistics and Medical Informatics, International School of Medicine Kavacik Güney Yerleskesi, Istanbul Medipol University, Istanbul, Turkey; 2 Department of Evidence for Population Health Unit, School of Epidemiology and Health Sciences, The University of Manchester, Manchester, United Kingdom; 3 Department of ENT, Medipol International School of Medicine, Istanbul Medipol University, Istanbul, Turkey; 4 Department of Neurology, School of Medicine, Istanbul Medipol University, Istanbul, Turkey

**Keywords:** hearing loss, stroke, cigarette smoking, alcohol use, vitamin D, obesity, sleepiness, mental heal

## Abstract

**INTRODUCTION:**

This study aimed to navigate the relationship between cigarette smoking, alcohol consumption, sleeping disorder, mental health and hearing loss in stroke patients.

**METHODS:**

This was a cross-sectional study that involved 1040 male and female patients aged 25–65 years. The research utilized physical examinations, radiological assessments, biochemical tests, and pure-tone audiometry (PTA) to evaluate hearing function.

**RESULTS:**

Among the 1040 stroke patients, 219 cigarette smokers (21.6%) were found to have hearing loss. Remarkable dissimilarities were observed in three categories: cigarette smokers with hearing loss, cigarette smokers without hearing loss, and non-smokers without hearing loss. These differences were noted in BMI (p<0.001), physical activity (p=0.002), hypertension (p<0.001), MP3 use (p<0.001), tinnitus (p<0.001), vertigo (p<0.001), dizziness (p<0.001), and headaches/migraines (p<0.001). Similarly, significant differences were identified among cigarette smokers with hearing loss, cigarette smokers without hearing loss, and non-smokers in relation to age (p<0.001), BMI (p<0.001), MP3 use (p=0.004), hypertension (p=0.028), ATP III metabolic syndrome (p<0.001), IDF metabolic syndrome (p<0.001), tinnitus (p<0.001), vertigo (p<0.001), dizziness (p=0.012), headaches/migraines (p<0.001), vitamin D (p<0.001), calcium (p<0.001), magnesium (p<0.001), potassium (p=0.019), fasting glucose (p<0.001), hemoglobin A1c (p<0.001), high blood pressure (p<0.001), microalbuminuria (p<0.001), and sleepiness (p=0.014). Multivariate stepwise regression analysis of cigarette smokers among stroke patients showed that vertigo (p<0.001), obesity (p<0.001), vitamin D deficiency (p<0.001), ATP III metabolic syndrome (p<0.001), IDF metabolic syndrome (p=0.004), calcium levels (p=0.008), headaches/migraines (p=0.039), and hypertension (p=0.025) could predict hearing loss.

**CONCLUSIONS:**

This study puts forward that smoking cigarettes along with factors like hypertension, obesity, vitamin D deficiency, and sleepiness, serve as notable danger element for loosing hearing ability among stroke patients. There is increasing evidence linking cigarette tobacco smoking to lung cancer and various adverse health effects.

## INTRODUCTION

Cigarette smoking poses a major global public health concern worldwide^1-5^. Several investigations have highlighted the interrelatedness of smoking and hearing loss^1,4,6^. It has been observed that smokers have a greater likelihood of experiencing hearing loss compared to non-smokers^6-8^. The auditory system is particularly vulnerable to the harmful effects of smoking^6-10^. The impact of both cigarette and waterpipe (hookah) smoking on the loss of hearing ability is extensively documented in the literature^1,6-10^.

The literature states that cigarette smoking is the primary risk factor for tracheal, bronchus, or lung (TBL) cancer, incidence rate of 27.7 per 100000 people, accounting for 80–90% of cases in regions with high smoking prevalence^5^. While other risk factors exist, smoking is the dominant driver of TBL cancer in many communities. The incidence of TBL-related cancers can potentially be predicted based on smoking rates.

Several studies have established a link between elevated long-term blood pressure and hearing loss^8,10-13^. More recent research has also found associations between hearing impairments and conditions such as hypertension^8,9-13^, stroke^10,14-16^, sleepiness^8,17^, and cigarette smoking^1,3-6,12^. However, the precise cause of hearing loss in many stroke patients who smoke cigarettes or waterpipes, remains unidentified^8,12,16^. Literature findings are showing that a reduction in hearing could significantly happen due to high threshold vitamin D levels^17-23^, making vitamin D levels a critical consideration in diet. Additionally, MP3 players have been identified as a remarkable danger for hearing loss^24^. This study aimed to explore the relationship between cigarette smoking, alcohol consumption, sleeping disorder, mental health and hearing loss among stroke patients.

## METHODS

### Participants

This cross-sectional study was carried out at the outpatient clinics of the Ear, Nose, and Throat (ENT) and Neurology Departments at Istanbul Medipol University Hospitals from January 2024 to March 2025. With a prevalence of 20% for smokers with hearing loss, a 99% confidence interval, and a 3% margin of error^12,16^, the calculated sample size was 1352 subjects, of which 11140 agreed to participate (response rate of 75%). Ethical approval was obtained from the Istanbul Medipol University Institutional Review Board (IRB) committee (IRB: E-10840098-772.02-1411 and IRB: E-10840098-202.3.02-7291).

### Design and smoking history

Smoking was defined as having smoked at least 100 cigarettes in a lifetime and currently smoking. The study compared smokers with and without hearing loss to non-smokers without hearing loss.

### Blood pressure and vitamin D assessment

Hypertension was assessed based on ≥140 mmHg systolic blood pressure (SBP) or ≥90 mmHg diastolic blood pressure (DBP), or regular antihypertensive medication use^16,19^. Serum 25(OH)D (25-hydroxy vitamin D) levels were measured using a competitive radio-immunoassay (RIA), a sensitive immunoassay technique involving antigen-antibody interaction *in vitro*, aided by radioisotopes (DiaSorin, Stillwater, Minnesota). Participants were categorized into three groups: those with vitamin D deficiency, those with insufficiency, and those with normal/optimal levels^16,19,22-23^.

### Hearing assessment

Hearing sensitivity was assessed using pure-tone audiometry (PTA) with two clinical digital audiometers (Interacoustics AC40 Clinical Audiometer and Garson Stadler GSI 61). Hearing loss was classified as normal (<26 dB) or impaired (≥26 dB)^8,12,16,19,21^.

### Patients Heath Questionnaire - Depression (PHQ-15)

The PHQ-15 is depression disorder screening. The Turkish version of the PHQ-15 has shown satisfactory reliability, with a Cronbach’s alpha of 0.79, pointing fine internal consistency. The total score ranges from 0 to 30, categorized as follows: 0–4 (none), 5–9 (mild), 10–14 (moderate), and 15–30 (severe). A score of 10 is considered the recognized cutoff value for identifying potential depressive symptoms.

### Sleepiness assessment

The Epworth Sleepiness Scale (ESS)^8,25^ was employed to examine sleepiness symptoms. This scale includes eight items evaluating the likelihood of dozing in specific situations (e.g. watching TV, sitting, talking) and is rated from 0 (would never doze) to 3 (high chance of dozing). Total scores span from 0 to 24, categorized into normal (0–7), mild (8–9), moderate (10–15), and severe (16–24) sleepiness^8,25^.

### Depression Anxiety Stress Scale (DASS-21)

The 21-item DASS-21, developed by Lovibond and Lovibond (1995), is used to assess anxiety, depression and stress. In the current study, the internal consistency was high, with Cronbach’s alpha values of α=0.85 for the depression sub-scale, α=0.83 for the anxiety sub-scale, and α=0.81 for the stress sub-scale. Stress was categorized as five levels: normal, mild, moderate, severe, and very severe.

### Statistical analysis

Data analysis was performed using SPSS software, version 25. The chi-squared test was applied to evaluate significant differences across categorical variables. To contrast these three group’s means, a one-way analysis of variance (ANOVA) was employed. Additionally, a multivariate stepwise linear regression analysis was conducted to examine the relationship between smoking and hearing loss in stroke patients, considering p<0.05 as significant.

## RESULTS

Out of 1140 patients studied, 19.2% were cigarette smokers who experienced hearing loss, while 10.9% were waterpipe smokers who have lost their hearing ability. Table 1 outlines the sociodemographic and clinical characteristics among the three groups: cigarette smokers with hearing loss, cigarette smokers without hearing loss, and non-smokers who did not use waterpipes and had no hearing loss. These dissimilarities were noted in BMI (p<0.001), physical activity (p=0.002), hypertension (p<0.001), MP3 player usage (p<0.001), tinnitus (p<0.001), vertigo (p<0.001), dizziness (p<0.001), and headaches/migraines (p<0.001).

**Table 1:** The general characteristics and clinical comparison of cigarette smokers with hearing loss versus without, among stroke patients (N=1140)

*Characteristics*	*Cigarette smokers with hearing loss (≥26 dB) n (%)*	*Cigarette smokers without hearing loss (<26 dB) n (%)*	*Non-smokers of cigarettes and waterpipe without hearing loss (<26 dB) n (%)*	*p*
**Total**, n	219	113	808	
**Age** (years)				
<40	72 (32.9)	15 (13.3)	272 (33.7)	
40–50	54 (24.7)	44 (24.9)	199 (24.9)	0.001
>50	93 (42.5)	54 (41.7)	337 (41.7)	
**Gender**				
Male	89 (40.6)	42 (37.2)	327 (40.5)	0.780
Female	130 59.4)	71 (62.8)	481 (59.5)	
**Marital status**				
Single	15 (6.8)	30 (50.0)	38 (4.8)	0.353
Married	204 (93.2)	13 (18.1)	769 (95.2)	
**Occupational status**				
Housewife	17 (9.4)	4 (4.7)	82 (9.4)	
Sedentary	80 (44.4)	48 (55.8)	379 (49.4)	
Manual	36 (20.0)	16 (18.6)	203 (23.2)	0.163
Businessman	25 (13.9)	8 (10.5)	142 (16.2)	
Arm/police/security officer	22 (12.2)	9 (10.5)	68 (7.8)	
**Income**				
Low	70 (32.0)	43 (38.1)	294 (36.9)	
Medium	78 (35.6)	38 (33.6)	303 (37.5)	0.386
High	71 (32.4)	42 (28.2)	211 (26.1)	
**Body mass index**				
Normal	63 (28.3)	8 (31.9)	270 (36.9)	
Overweight	84 (38.4)	17 (50.0)	335 (38.5)	0.001
Obese	72 (32.9)	88 (18.1)	203 (24.6)	
**Physical exercise** (Yes)	41 (18.7)	33 (29.2)	247 (30.6)	0.002
**MP3 player use** (Yes)	191 (87.2)	97 (75.0)	610 (85.7)	0.004
**Do you hear TV sounds** (Yes)	198 (90.4)	101 (89.4)	700 (86.6)	0.270
**Family history of hypertension** (Yes)	57 (26.0)	18 (15.9)	98 (12.1)	0.001
**Family history of diabetes** (Yes)	59 (26.9)	21 (18.7)	95 (11.8)	0.001
**ATP III metabolic syndrome** (Yes)	74 (13.8)	8 (8.5)	111 (17.6)	0.242
**IDF metabolic syndrome** (Yes)	31 (15.6)	10 (10.6)	127 (20.2)	0.335
**Tinnitus** (Yes)	111 (50.7)	10 (10.6)	93 (11.5)	0.001
**Vertigo** (Yes)	127 (58.0)	13 (11.5)	101 (12.5)	0.001
**Dizziness** (Yes)	63 (28.8)	28 (23.8)	118 (14.6)	0.001
**Headache and migraine** (Yes)	86 (39.3)	29 (25.7)	121 (10.0)	0.001

Table 2 presents the prevalence of mental health symptoms and sleep disorders. It shows that 10% of individuals with sleeping disorders had PHQ-15 scores ≥10, compared to 5% of those without sleeping disorders. The data indicate significant differences between those with and without sleep disorders, with higher prevalences of PHQ-15 scores ≥10 (p<0.001), depression (p<0.001), anxiety (p=0.002), and stress (p<0.001) among individuals with mental health conditions.

**Table 2:** Prevalence of mental health symptoms by gender (N=1140)

*Variables*	*Cigarette smokers with hearing loss (≥26 dB) n (%)*	*Cigarette smokers without hearing loss (<26 dB) n (%)*	*Non-smokers of cigarettes and waterpipe without hearing loss (<26 dB) n (%)*	*p*
**Total**, n	219	113	808	
**GHQ-15**				
None	58 (26.5)	55 (487)	373 (46.2)	
Mild	56 (25.6)	11 (9.7)	141 (17.5)	
Moderate	44 (20.1)	20 (17.7)	140 (17.3)	0.001
Severe	61 (27.9)	27 (23.9)	154 (19.1)	
**Epworth sleepiness scale**				
Normal	108 (49.3)	68 (60.2)	465 (57.5)	
Mild	53 (24.2)	26 (23.0)	124 (15.3)	0.013
Moderate	46 (21.0)	16 (14.2)	175 (21.7)	
Severe level	12 (5.5)	3 (2.7)	44 (5.4)	
**DASS21 depression**				
Normal	24 (11.0)	39 (34.5)	221 (27.4	
Mild	43 (19.6)	23 (20.4)	207 (25.2)	
Moderate	66 (30.1)	25 (21.9)	172 (21.3)	0.001
Severe	45 (20.5)	18 (15.9)	146 (18.1)	
Extreme	41 (18.7)	8 (7.1)	62 (7.7)	
**DASS21 anxiety**				
Normal	58 (26.5)	24 (21.2)	287 (35.5)	
Mild	70 (32.0)	40 (35.4)	284 (35.1)	
Moderate	29 (13.2)	15 (13.3)	83 (10.3)	0.002
Severe	39 (14.6)	21 (18.6)	77 (9.5)	
Extreme	30 (13.7)	13 (11.5)	77 (9.5)	
**DASS21 stress**				
Normal	85 (29.7)	39 (34.5)	258 (33.6)	
Mild	36 (16.4)	30 (26.5)	164 (20.3)	
Moderate	31 (14.2)	20 (17.7)	134 (17.4)	0.001
Severe	43 (19.6)	13 (11.5)	137 (14.5)	
Extreme	44 (20.1)	11 (9.7)	79 (9.8)	

Table 3 displays the clinical biochemical values for cigarette smokers with hearing loss among three stroke groups, showing differences in vitamin D (p<0.001), hemoglobin (p<0.001), calcium (p=0.004), magnesium (p<0.001), fasting glucose (p<0.001), HbA1C (p<0.001), HDL (p=0.009), microalbuminuria (p<0.001), systolic BP (p<0.001), and diastolic BP (p<0.001). Sleepiness was notably higher among cigarette smokers with hearing loss compared to non-smokers without hearing loss (p=0.013).

**Table 3:** Clinical biochemical variables comparison cigarette smoker with hearing loss patients versus without among stroke patients (N=1140)

*Variables*	*Cigarette smokers with hearing loss (≥26 dB) Mean ± SD*	*Cigarette smokers without hearing loss (<26 dB) Mean ± SD*	*Non-smokers of cigarettes and waterpipe without hearing loss (<26 dB) Mean ± SD*	*p p-p-value significance*
**Total**, n	219	113	808	
Vitamin D (ng/mL)	16.99 ± 8.07	19.88 ± 8.51	21.68 ± 9.15	<0.001
Hemoglobin (g/dL)	12.78 ± 1.67	13.76 ± 1.63	14.04 ± 1.96	<0.001
Magnesium (mmol/L)	0.66 ± 0.08	0.78 ± 0.08	0.97 ± 0.09	<0.001
Potassium (mmol/L)	3.58 ± 0.80	3.55 ± 0.69	3.53 ± 0.49	0.243
Calcium (mmol/L)	1.94 ± 0.10	1.96 ± 0.11	1.98 ± 0.10	0.009
Phosphorous (mmol/L)	1.27 ± 0.27	1.26 ± 0.34	1.27 ± 0.31	0.966
Creatinine (mmol/L)	75.47 ± 15.34	75.47 ± 15.34	74.22 ± 14.16	0.643
Fasting Glucose (mmol/L)	7.29 ± 1.46	7.29 ± 1.46	6.30 ± 1.34	<0.001
HbA1c	7.32 ± 1.36	6.50 ± 1.16	6.29 ± 1.01	<0.001
Cholesterol (mmol/L)	4.73 ± 0.86	4.71 ± 0.86	4.80 ± 0.88	0.265
HDL (mmol/L)	1.04 ± 0.22	1.09 ± 0.35	1.11 ± 0.31	0.020
LDL (mmol/L)	1.82 ± 0.68	1.971 ± 0.83	1.89 ± 0.83	0.324
Albumin (mmol/L)	41.88 ± 3.85	41.48 ± 4.12	42.28 ± 5.10	0.179
Bilirubin (mmol/L)	7.59 ± 2.60	6.99 ± 2.12	7.33 ± 2.62	0.130
Triglyceride (mmol/L)	1.66 ± 0.69	1.66 ± 0.66	1.64 ± 0.71	0.958
Ferritin (mmol/L)	250.23 ± 39.72	174.72 ± 22.86	227.21 ± 32.20	0.179
Uric acid (mmol/L)	273.89 ± 2.56	294.35 ± 58.31	283.49 ± 67.60	0.023
Systolic BP (mmHg)	144.00 ± 7.63	143.00 ± 7.63	127.00 ± 3.90	<0.001
Diastolic BP (mmHg)	87.89 ± 8.33	87.89 ± 8.33	79.15 ± 7.31	<0.001
Microalbuminuria	11.78 ± 3.25	11.75 ± 3.35	8.69 ± 1.65	<0.001
	***n (%)***	***n (%)***	***n (%)***	
**Vitamin D level** (25(OH)D ng/mL)				
Deficiency level (<20)	151 (68.9)	136 (68.3)	408 (50.2)	
Insufficiency level (20–29)	36 (16.4)	35 (17.6)	260 (32.2)	<0.001
Optimal level (>30)	32 (14.6)	28 (14.1)	142 (17.6)	

Table 4 outlines the multivariate regression analysis for predicting risk factors in cigarette smokers with hearing loss among stroke patients, identifying vertigo (p<0.001), obesity (p<0.001), vitamin D deficiency (<20 ng/mL) (p<0.001), ATP III Metabolic Syndrome (p<0.001), IDF Metabolic Syndrome (p=0.004), calcium (mmol/L) (p=0.008), headaches/migraines (p=0.039), and hypertension (p=0.025) as significant predictors among stroke patients.

**Table 4:** Multivariate stepwise regression analysis for predictors of hearing impairment associated with cigarette smoking in stroke patients (N=1140)

*Independent variables*	*B*	*SE*	*β*	*t-test*	*p*
Vertigo (Yes)	0.423	0.035	0.325	11.974	<0.001
Obesity	0.015	0.003	0.134	5.039	<0.001
Vitamin D deficiency (<20 ng/mL)	0.006	0.002	0.101	3.802	<0.001
ATP III metabolic syndrome	0.170	0.049	0.117	3.476	<0.001
IDF metabolic syndrome	0.131	0.045	0.097	2.918	0.004
Calcium (mmol/L)	0.332	0.124	0.071	2.672	0.008
Headache and migraine (Yes)	0.073	0.035	0.056	2.064	0.039
Hypertension (Yes)	-0.070	0.031	-0.064	-2.251	0.025

SE: standard error.

## DISCUSSION

This study revealed a significant association between cigarette smoking, waterpipe use, and hearing loss among stroke patients, consistent with previous studies^3-10,12^. Exploring the factors related to smoking and hearing loss in stroke patients is crucial for preventing hearing loss. Reducing or quitting smoking could potentially prevent hearing issues^3-10,12^. Hu et al.^5^ discovered that individuals who had quit smoking faced a lower risk of hearing loss compared to those who continued to smoke. Recently, Bener et al.^1,12,16^ confirmed the link between cigarette smoking and hearing ability deprivation among hypertension and stroke patients, aligning with this study’s findings.

This study also identified a strong correlation between obesity, comorbidities, and diminished hearing in cigarette as well waterpipe smokers, which is consistent with previous findings^8,12-16,20-21^. The results of this study align with previous research concerning stroke, hypertension, and tinnitus^2-4,8,9,12-13^. Latest literature findings have indicated that hearing deterioration is rather prevalent among hypertension and diabetes patients in contrast to the healthy population^1-14-16,20^.

The current survey indicated a notable positive association between hearing disease and obesity, which is in line with previously reported studies^1,8,12,22^. The popularity of MP3 players for listening to music, as noted in this study, is consistent with another research^24^.

A recent study^5^ reported significant variations in the burden of tracheal, bronchus, and lung (TBL) cancer across 204 countries and territories. While global age-standardized incidence, death, and disability-adjusted life years (DALY) rates are declining, some countries still show increasing trends in these rates.

Research by Dawes et al.^11^, Bener et al.^1,16,19^, and Bigman^20^ suggested that increasing vitamin D levels can reduce hearing problems. Additionally, vitamin D deficiency has been linked to both low and high-frequency hearing loss in adults and the elderly^18-20^. These findings align with the present study. Vitamin D performs a key role in the human auditory system, and its deficiency negatively affects the ears, particularly the inner ear^18-20^. Overall, this study found that hypertension, vitamin D deficiency, and sleepiness were potential risk factors for hearing loss among smokers, suggesting the need for immediate prevention strategies through media campaigns. Some studies have also observed a relationship between sleep disorders, hearing loss, and tinnitus^26^.

### Limitations

The study has some limitations. First, the cross-sectional design does not allow for determining causal relationships. Second, some patients were excluded due to the time required for audiometric assessments. Third, the smoking information was self-reported, which may introduce bias. Fourth, the sleepiness data obtained from the Epworth scale is subjective and may not be entirely accurate.

## CONCLUSIONS

This study puts forward that smoking cigarette along with factors like hypertension, obesity, vitamin D deficiency, and sleepiness, serve as notable risks for loosing hearing ability among stroke patients. There is increasing evidence linking cigarette tobacco smoking to lung cancer and various adverse health effects.

## Data Availability

The data sets used and/or analyzed in this study are available from the corresponding author on reasonable request.
